# Curiosity Killed the Cat but Not Memory: Enhanced Performance in High-Curiosity States

**DOI:** 10.3390/brainsci12070846

**Published:** 2022-06-28

**Authors:** Caterina Padulo, Erika Marascia, Nadia Conte, Noemi Passarello, Laura Mandolesi, Beth Fairfield

**Affiliations:** 1 Department of Psychological, Health and Territorial Sciences, University of Chieti, 66100 Chieti, Italy; caterina.padulo@unich.it (C.P.); nadia.conte@unich.it (N.C.); 2 Department of Humanities, University of Naples, Federico II, 80133 Naples, Italy; noemi.passarello@unina.it (N.P.); laura.mandolesi@unina.it (L.M.); beth.fairfield@unina.it (B.F.)

**Keywords:** trivia, aging, memory, emotion

## Abstract

Curiosity benefits memory for target information and may also benefit memory for incidental information presented during curiosity states. However, it is not known whether incidental curiosity-enhanced memory depends on or is affected by the valence of the incidental information during curiosity states. Here, older and younger participants incidentally encoded unrelated face images (positive, negative, and neutral) while they anticipated answers to trivia questions. We found memory enhancements for answers to trivia questions and unrelated faces presented during high-curiosity compared with low-curiosity states in both younger and older adults. Interestingly, face valence did not modify memory for unrelated faces. This suggests processes associated with the elicitation of curiosity enhance memory for incidental information instead of valence.

## 1. Introduction

Some information is easier to remember than other information and one of the reasons may be linked to curiosity. Curiosity is an intrinsic motivation to gain knowledge for its own sake rather than its instrumental utility [1,2,3] and is an essential component of human activity and information processing. In fact, individuals generally spend much time each day searching for information that interests them through the internet, books, asking questions, etc. According to Kashdan et al. [4], curiosity is defined as “the recognition, pursuit and desire to explore novel, uncertain, complex, and ambiguous events,” which initiates and facilitates learning. In addition, being curious and knowledge seeking has been associated with a number of positive outcomes, including better physical, psychological, cognitive, and social well-being. For example, studies show that curiosity can influence encoding and retrieval of information [5] and may also have broader effects that extend to memory for implicitly encoded unrelated information encountered in close temporal proximity to interesting information through activity in the midbrain and hippocampus and by functional connectivity between these regions [6]. These findings suggest that there may be a link between the mechanisms supporting extrinsic reward motivation and intrinsic curiosity and highlight the importance of stimulating curiosity to create more effective learning experiences. According to the PACE framework of how curiosity shapes learning and memory, dopaminergic functions improve hippocampus-dependent coding and memory consolidation [7].

Interestingly, although curiosity is a fundamental part of human motivation, supporting a variety of human intellectual behaviors ranging from learning in children to scientific discovery, few studies have focused attention on curiosity in aging populations. Previous studies that have examined subjective feelings of curiosity and aging suggest that normal aging leads to a decline in at least some aspects of curiosity. For example, Robinson et al. [8] found a decline from early to late adulthood in three distinct dimensions of curiosity: interpersonal curiosity, epistemic curiosity, and intrapersonal curiosity [9].

One explanation for this decline in curiosity may be linked to changes in motivation. The socioemotional selectivity theory [10] posits that people are motivated according to two broad goal categories. On the one hand, individuals seek to acquire knowledge, novelty, and to expand their knowledge. On the other, individuals seek to regulate negative and maintain positive and meaningful states. These goals operate across the entire lifespan and individuals are generally motivated to learn new knowledge and, later on, to maintain meaningful emotional states. Indeed, as individuals age, they perceive time as more limited and, as a result, become more selective when investing time and resources. Specifically, when time is perceived as unlimited, individuals are motivated to expand knowledge and skills in order to prepare for the future ahead. On the contrary, when time is perceived as limited, long-term expansive goals are less likely to be realized and people are more likely to shift attention to the present and to goals that grant more immediate satisfaction. Consequently, older adults may prioritize emotionally meaningful goals over knowledge-seeking goals and it is possible that, in general, older adults perceive curiosity as being less valuable.

Nonetheless, a number of recent studies suggests that curiosity may actually play a critical role in maintaining cognitive functioning, wellbeing, and physical health in older adults. For example, studies adopting trivia questions to investigate the effect of curiosity on memory for answers found that both younger [5,11,12,13] and older adults [14,15] show curiosity-driven memory advantages for high-curiosity trivia answers compared to low-curiosity ones. In addition, studies that presented temporally contiguous, unrelated information in the interval between the presentation of the trivia question and the answer, when curiosity is assumed to be at its peak [6,14,16,17], found better memory for the implicitly encoded information associated with high-curiosity trivia items compared to low, but only in younger adults [6,17]. Galli et al. [14], instead, found mixed results showing curiosity driven memory advantages for neutral faces in older adults in one study (Exp 1) [14] but not in a second study (Exp 2) [14]. Therefore, states of high curiosity seem to improve learning for topics that strike an individual’s curiosity but, at least in certain conditions, may also improve memory for information beyond the specific target of the individual’s curiosity [6,14,17,18]. However, it is not clear whether memory is enhanced for valenced incidental information during curiosity states as well.

Here, we aimed to further explore the effects of curiosity on long-term memory and incidental memory in healthy younger and older adults. To this end, we modified the paradigm adopted by Galli et al. [14] to include valenced faces (positive and negative), as well as neutral ones, to investigate curiosity and affective information interactions on memory performance. Emotional enhancement effects are widely demonstrated in the literature, with specific valence effects linked to age [19,20]. For example, both younger and older adults generally remember affective (positive and negative) information better than neutral information. Moreover, older adults often show positivity effects, that is, better memory for positive information compared to negative information, while younger adults seem to remember negative information better than positive information [21,22]. In line with studies in the literature, we expect younger and older adults to remember more trivia answers in high-curiosity states compared to low-curiosity states. Moreover, if curiosity is perceived as being less important by older adults compared to younger ones, we would expect younger adults to show even larger advantages for trivia answers in high-curiosity states compared to older adults. Regarding memory for incidental face encoding, we expect better memory for incidental faces in high curiosity than low curiosity in younger adults, since younger adults may consider curiosity more meaningful than valence. Instead, we expect older adults to be more sensitive to affective information, since valence is typically considered as more meaningful by them. If valence guides memory performance in older adults, we expect older adults to remember positive and negative faces better than neutral ones as well as typical positivity biases, that is, better memory for positive faces compared to negative ones [19].

## 2. Materials and Methods

### 2.1. Participants

A total of 45 younger adults (mean age = 26.38, SD = 4.44) and 45 community-dwelling older adults (mean age = 65.67, SD = 7.36) from Chieti and Pescara (Italy) took part in the study. Our sample size was imposed by resource constraints [23]. Before beginning the experimental session, all participants completed the forward and backward digit span of the Wechsler Adult Intelligence Scale-Revised (WAIS-R) [24], the Positive and Negative Affective Scale (PANAS) [25] to assess current mood, and the FAS [26] for verbal fluency. Older adults also completed the Mini Mental State Examination (MMSE) [27] to screen for general cognitive abilities. Neuropsychological and demographic data are reported in Table 1. Older and younger adults differed for years of education and verbal fluency. However, it should be noted that this is very typical of the older Italian population, and MMSE scores confirmed that older adults did not show significant cognitive impairments. All participants reported being in good physical and mental health, had no known memory deficits, and normal or corrected-to-normal visual acuity. Exclusion criteria included history of severe head trauma, stroke, neurological disease, severe medical illness, or alcohol or substance abuse. The study was approved by the Department of Psychological, Health and Territorial Sciences ethic committee and all participants gave written informed consent in accordance with the Declaration of Helsinki [28] prior to inclusion in the study.

### 2.2. Materials

We selected 104 trivia questions with corresponding answers from Galli et al. [14]. The questions and answers were translated into Italian and rated for levels of curiosity by an independent group of younger and older adults along a 6-point Likert scale. From these 104 trivia questions, we selected 72 so that the questions were equally distributed along curiosity levels.

We selected 108 faces from the FACES database [29]. Faces were colored, frontal-view pictures composed of 36 negative, 36 neutral, and 36 positive faces. For each valence, half of the faces were male and the other half female, and half were older adults and half were younger adults. Of the 108 faces, 72 (24 positive, 24 negative, and 24 neutral) were used for incidental encoding in the study phase while 36 (12 positive, 12 negative, and 12 neutral) were used as new items during test. We also used an additional 4 faces and 4 trivia questions and their answers for the practice session to allow participants to familiarize with the experimental procedure. These were not included in the experimental session. The match face/trivia was counterbalanced across subjects and presentation of the stimuli was randomized.

### 2.3. Procedure

#### 2.3.1. Study Phase

**Figure 1:**
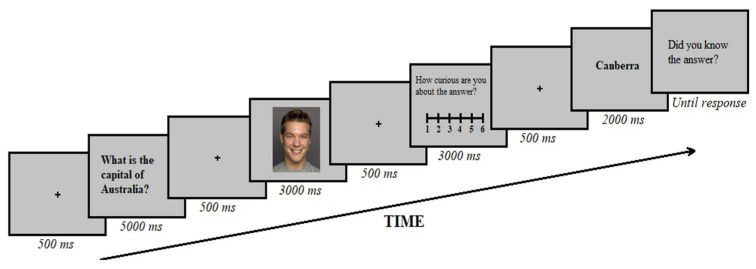
Schematic illustration of the experimental procedure.

The experiment was run using E-prime [30]. The study phase contained two separate blocks of 36 trials, 12 for each valence. Each trial began with a 500 ms fixation cross on a grey background, followed by the presentation of the trivia question for 5000 ms. After another 500-ms fixation cross, a face stimulus appeared, and participants were instructed to verbally judge whether the person depicted would know the answer to the trivia question to ensure that participants encoded face stimuli. Answers were collected by a researcher using a scoring protocol. When the slide displayed the question “How curious are you about the answer?”, participants rated their curiosity on a Likert scale from 1 to 6 (1 = not at all; 6 = very curious) using the keypad. After another 500-ms fixation cross, the trivia answer appeared for 2000 ms and participants indicated whether they already knew the answer to the trivia question (YES/NO) by pressing x or z on the keyboard (see Figure 1 for an example of a trial). The study phase lasted approximately 30 min and was followed by a 15-min rest interval before beginning the test phase.

#### 2.3.2. Test Phase

##### Trivia

Participants were given a list of all 72 trivia questions and asked to write down as many answers as they could remember from the study phase.

##### Faces

Participants were presented with 108 faces (72 old and 36 new) and instructed to indicate whether the face had been presented before (YES/NO) by pressing one of two keys on the computer keyboard (z = yes; x = no).

## 3. Results

Data analyses were carried out using Statistica 8.0 [31]. As conducted in previous studies [14], we considered ratings of 1, 2, and 3 as low curiosity and ratings of 4, 5, and 6 as high curiosity, and included in the analyses only trials for which participants indicated that they did not know the answer. For each subject, we calculated the percentage of correctly recalled answers and mean accuracy scores on face recognition, calculated as hits minus false alarm (FA), for all conditions. Thus, we ran two separate General Linear Models (GLMs), one for trivia recall and one for face recognition, with Curiosity (two levels: high and low) and Valence (three levels: negative, neutral, and positive) as within-subject factors and Group (two levels: younger and older) as a between-subjects factor.

### 3.1. Trivia Recall

**Figure 2:**
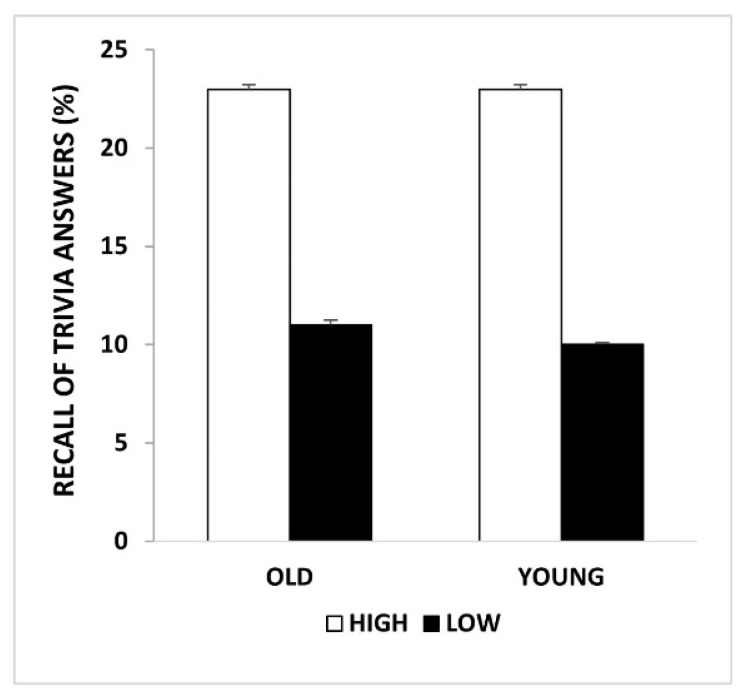
Recall of trivia answers: memory performance for levels of curiosity in young and older adults.

Analyses revealed no main effect of Group (*p* = 1.0), showing that recall of trivia answers was similar in the two groups, nor a main effect of Valence (*p* = 0.425). We found a significant main effect of Curiosity (F_(1,88)_ = 46.601, *p* < 0.001, η_p_^2^ = 0.346), showing that memory for trivia answers increased as levels of curiosity increased in both younger and older adults (Figure 2). The two-way and three-way interactions were not significant (all *p* ≥ 0.695).

### 3.2. Face Recognition

**Figure 3:**
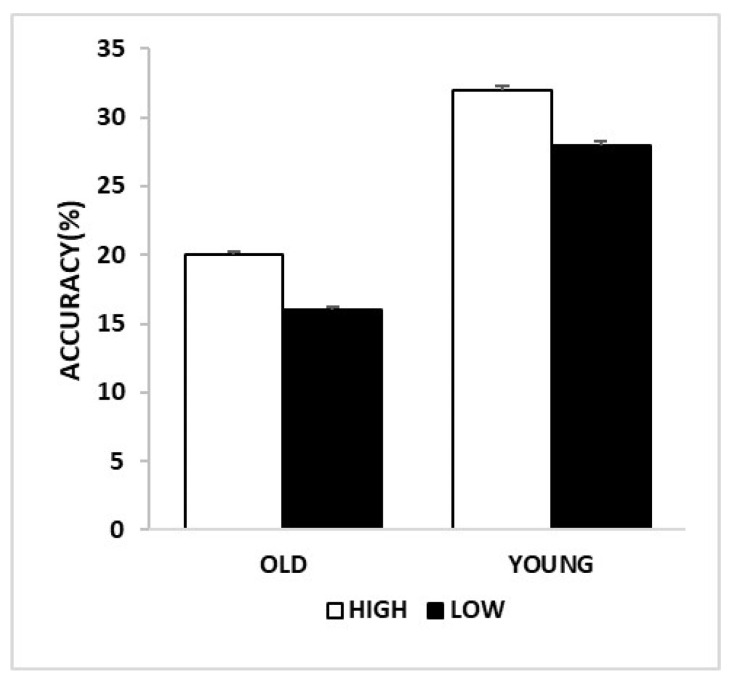
Accuracy for memory of faces: memory performance for levels of curiosity in young and older adults.

Analyses revealed a significant main effect of Group (F_(1,88)_ = 27.724, *p* <.001, η_p_^2^ = 0.240, power to detect the effect was 0.999), showing that accuracy was higher in younger compared to older adults. We found a main effect of Curiosity (F_(1,88)_ = 4.7339, *p* = 0.03, η_p_^2^ = 0.051, power to detect the effect was 0.576), indicating that accuracy improved as curiosity increased (Figure 3). We found no main effect of Valence (*p* = 0.368). The two-way and three-way interactions were not significant (all *p* ≥ 0.314).

## 4. Discussion

Although previous research suggests that normal aging is associated with reduced curiosity [8], curiosity may play an important role in maintaining cognitive function, and mental and physical health in older adults. In this study, we examined the effects of curiosity on long-term memory for trivia answers and implicit memory for positive, negative, and neutral faces in younger and older adults. In line with studies in the literature, we found that curiosity induced benefits in memory for trivia answers in younger [5,6,12,32] and older adults [14], suggesting that the beneficial effects of curiosity on memory are preserved during aging. Indeed, older adults benefited in a way that raised their performance to levels of younger adults. Moreover, memory for unrelated faces encountered during the study phase also benefited from curiosity [14].

Interestingly, we did not find valence-related benefits on memory performance, suggesting that curiosity may be an even more effective motivational boost for memory. Many studies on emotion and memory [21] have emphasized that, although older adults show declines in cognitive control, emotion regulation abilities and memory for affective information seem to remain intact. In fact, age does not seem to impair affective information processing and results consistently show how younger and older adults remember affective information better than neutral information [20]. Furthermore, many studies have revealed interesting age-related memory patterns linked to the valence of emotional information [21]. In particular, these studies show how older adults generally remember positive information (positivity effect) better than negative and neutral information [19,33,34,35].

There may be several possible reasons for why we did not find valence effects on incidental face memory. First, the incidental face stimulus and the trivia answer were separated by an evaluation task (“Do they know the answer?”). It may be that focusing attention on this task may have disrupted or “covered” valence effects. Indeed, the effect of curiosity on incidental face memory seems to depend on curiosity satisfaction [12], surprise [36], or post-answer interest [15] and may have driven participants to follow knowledge-seeking motivational behaviors rather than meaningful motivational behaviors. Moreover, we cannot exclude arousal effects that may have hidden valence effects. In fact, we selected faces from the FACES database [29], which does not include specific rating information regarding arousal levels.

Moreover, according to the motivational perspective offered by SST, positivity biases are the result of age-related shifts in goal priorities that increase the salience of emotionally gratifying information in attention and memory. Since cognitive resources are required to direct information processing toward task-relevant stimuli and away from less relevant stimuli [37], it may be that, in tasks where information processing is highly relevant, older adults may direct resources away from affective stimuli [38], thus eliminating specific valence effects.

In addition, positivity in attention supports chronically activated goals about emotional meaning and satisfaction that older adults tend to prioritize [39,40]. Theoretically, the positivity effect reflects an adaptive accommodation of goal-directed cognitive processing. In our study, face encoding was incidental, and participants were not told that memory for faces would be tested. This may have led older adults, who have limited cognitive resources, to focus available cognitive resources on the explicit task [41].

Our study, however, is not without limitations. Although our results show evidence of a beneficial effect of curiosity on memory, other factors may be responsible for memory enhancement. For example, memory benefits could result from an increase in attention or arousal associated with the trivia questions selected for our task, rather than from an increase in curiosity per se. Moreover, benefits could also result from an increase in tip-of-the-tongue experiences, again associated with the trivia questions used to elicit curiosity [42]. Memory benefits could also be influenced by variables that are conceptually overlapping with curiosity, such as need for cognition, interest, or level of difficulty.

Future studies could improve upon the current design by including measures of attention and arousal, such as eye-tracking and/or biofeedback, or by directly manipulating curiosity and similar constructs within the same experimental design.

## 5. Conclusions

Although normal aging is associated with reduced curiosity, curiosity may play an important role in maintaining cognitive functioning, and mental and physical health. In this study, we found that both memory for trivia answers and memory for unrelated faces benefited from curiosity. These results suggest that researchers need to consider motivational factors, especially curiosity, to better understand age-related memory performance. Future studies should also examine the role of curiosity on incidental memory for a broader range of stimuli. The impact of such work could be applicable to a wide range of areas and would need to be tested in the real world. This may be particularly important given the actual pandemic situation in which we need to rapidly disseminate policies and educate the public on disease and health. Identifying ways to arouse people’s curiosity in order to optimize learning and wellbeing may also have practical implications for policies and interventions for successful aging.

## Figures and Tables

**Table 1:** Neuropsychological and demographic data.

	Younger = 45 (31 F)		Older = 45 (21 F)	
	M	SD	M	SD
Age group	26.34	4.49	65.67	7.36
Years of education	16.27 ***	2.67	13.08	5.09
PANAS pos	32.52	7.09	31.66	5.76
PANAS neg	22.82	6.89	17.30	5.08
Digit Span Forward	7.68	2.26	6.82	1.69
Digit Span Backward	6.95	2.31	5.24	1.84
FAS	36.41 ***	8.61	28.79	6.53
MMSE			29	1.17

Note: Positive and Negative Affect Schedule [25]; Digit Span-Forward and the Digit Span-Backward [24]; FAS [26]; and Mini Mental State Examination [27]. *** = *p* < 0.001.

## Data Availability

Not applicable.
